# Energy Release Characteristics and Reaction Mechanism of PTFE/Al/Bi_2_O_3_ Reactive Materials under Drop-Hammer Test

**DOI:** 10.3390/polym14071415

**Published:** 2022-03-30

**Authors:** Chunlan Jiang, Rong Hu, Liang Mao, Zaicheng Wang, Wenyu Xu, Wanxiang Hu

**Affiliations:** 1 State Key Laboratory of Explosion Science and Technology, Beijing Institute of Technology, Beijing 100081, China; jiangchunwh@bit.edu.cn (C.J.); 3120205127@bit.edu.cn (R.H.); wangskyshark@bit.edu.cn (Z.W.); alpha_direwolf@163.com (W.X.); 13940156612@163.com (W.H.); 2 Jiangsu Shuguang Guangdian Co., Ltd., Yangzhou 225009, China

**Keywords:** PTFE-based reactive materials, content of Bi_2_O_3_, shock-induced chemical reaction, drop-hammer test

## Abstract

To obtain the influence of the Bi_2_O_3_ particle content of a PTFE/Al/Bi_2_O_3_ reactive material (later referred to as PAB) on its shock-induced chemical reaction (SICR) characteristics, five kinds of PAB with different Bi_2_O_3_ contents were prepared; the reaction process in a drop-hammer test, recorded using a high-speed camera, was analyzed. The ignition and reaction mechanisms of PAB under mechanical impact were analyzed based on the thermochemical reaction characteristics and the microstructure. The results show that with an increase in Bi_2_O_3_ content, the shock-induced chemical reaction duration and the sensitivity of PAB increase, and then decrease. When the Bi_2_O_3_ content is 9%, the impact sensitivity is the highest and the reaction duration is the longest. The heating at the crack tip is responsible for PAB ignition under long-pulse low-velocity impact. During ignition, PAB undergoes several physicochemical changes such as the melting of PTFE, a PTFE/Bi_2_O_3_ reaction, an Al/Bi_2_O_3_ reaction, pyrolysis of the melted PTFE, and a C_2_F_4_/Al reaction; moreover, the presence of Bi_2_O_3_ decreases the excitation threshold of the reactive material, which facilitates the propagation of the reaction and improves the degree of the reaction and overall energy release of the reactive material.

## 1. Introduction

Reactive material has attracted much attention in recent years as new type of energetic material, which usually behaves with less sensitivity and does not react spontaneously, but releases large amounts of chemical energy under intense impact loads and produces explosive or deflagration effects. Metal polymers, especially PTFE/Al reactive materials, have become an important research field due to their unique energy-release properties [1,2,3]. However, PTFE/Als’ low material density and mechanical strength limit their applicability as functional parts. Nowadays, the design of new reactive materials with good energy release and the investigation of the impact-reaction phenomena and mechanisms of new reactive materials have become important directions for research in this field.

Thermite, as a traditional energetic material, consists of Al powder mixed with metal oxides or non-metal oxides with strong oxidability. Thermite can undergo violent redox reactions and release large amounts of heat when driven by heat or mechanical forces. Furthermore, its high energy density and good safety make it a promising material [4]. Since PTFE/Al and thermite have the same reaction component metal, i.e., Al, the combination of PTFE/Al reactive materials with thermite is a new approach to improving the energy release characteristics of PTFE/Al-based reactive materials [5,6,7]. Therefore, the formulations and preparation processes for obtaining ternary reactive materials that can initiate thermite reactions and have unique impact reaction properties have become a hot topic [8,9,10,11].

Bismuth oxide (Bi_2_O_3_), one of the essential bismuth compounds, has a density of 8.9 g/cm^3^, a melting point of 820 °C, and a boiling point of 1890 °C. Since the boiling point of bismuth metal is only 1564 °C, which is much lower than other metals and lower than the maximum reaction temperature of thermite (about 3000 °C), there is a large amount of bismuth gas produced during the thermite reaction [12,13,14,15,16]. The higher reaction heat and large amounts of gaseous products give Al/Bi_2_O_3_ thermite a good work capacity. When applied to reactive materials, it may also improve the destructive power of implosion. Due to the excellent properties of Bi_2_O_3_ in many aspects such as density, gas production, and energy density, the ternary reactive material system PAB was constructed, which enabled the reactive material to exhibit better energy release of the reactive material and solve the practical engineering application problems. 

The study of PAB is just beginning. Lan Jia [17] tested the reaction energy and combustion rate of various ternary reactive materials and filmed the shock-induced chemical reaction process of reactive materials under the split Hopkinson pressure bar test with a high-speed camera. The test results showed that PAB had the best combustion performance, prolonged reaction duration, and a significant gas production rate. Yuan Ying [18,19] studied and established a prediction model for the impact sensitivity of PAB. Subsequently, the reaction behavior of functional gradient materials composed of PAB was studied using ballistic impact tests, and the enhanced energy release performance of PTFE-based materials by Bi_2_O_3_ was confirmed.

In general, PAB has not been sufficiently studied, especially in terms of its shock-induced chemical reaction characteristics, mechanism of ignition, and propagation of reaction. In this paper, PABs with different Bi_2_O_3_ contents were prepared using cold pressing and sintering, and the effect of Bi_2_O_3_ content on the energy release characteristics of PAB under mechanical impact loading with a drop hammer was studied. With the help of differential scanning calorimetry (DSC); thermos-gravimetric analysis (TG); X-ray diffraction (XRD); the scanning electron microscopy and energy dispersive method (SEM-EDS); and the hot-spot generation and ignition mechanism, the multi-component physicochemical reaction process and propagation of the reaction in PAB were investigated from multiple perspectives, which fully illustrated the ignition and reaction mechanism of PAB under mechanical impact. The results of this study are of great reference significance for the study of shock-induced chemical reactions in PAB- and PTFE-based reactive materials.

## 2. Materials and Methods

### 2.1. Formulations and Preparation

Both PTFE and Bi_2_O_3_ can undergo a violent redox reaction with Al, calculated using the following reaction equation:

4Al + 3C_2_F_4_ = 4AlF_3_+ 6C (1)

(2) $${2\mathrm{Al}+\mathrm{Bi}}_{2}O_{3}{=\mathrm{Al}}_{2}O_{3}+2\mathrm{Bi}$$

Based on the chemical reaction equation, the mass ratio of PTFE and Al in the PTFE/Al reaction is 74:26, and the mass ratio of Bi_2_O_3_ and Al in the Al/Bi_2_O_3_ reaction is 11:89. Considering that a higher content of particles within the composite system will be detrimental to the mechanical properties and formability of the composite, Al/ Bi_2_O_3_ additions with mass fractions of 5%, 10%, 15% and 20% (11:89 mass ratio) were added to the stoichiometric ratio of PTFE/Al (later referred as PA). The formulations are shown in Table 1.

**Figure 1:**
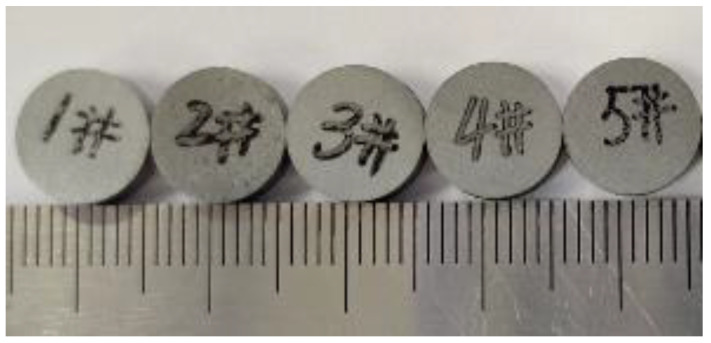
PAB specimens.

The specimens were prepared using the molded sintering method, and the raw materials were PTFE powder with an average particle size of 34 μm (7A, DuPont, Wilmington, DE, USA), high-purity spherical aluminum powder with an average particle size of 10 μm, and spherical Bi_2_O_3_ powder with an average particle size of 2–3 μm (Tianjiu Metal materials Co., Ltd., Changsha, China). The prepared PAB materials were processed into a circular disk-shaped specimen (ϕ10 mm × 3 mm), with a good surface finish and parallelism, for the drop-hammer test; the specimens are shown in Figure 1.

The presence of pores in the reactive materials prepared by cold pressing and sintering was inevitable, and the relative densities were calculated. The calculated results are shown in Table 2. As seen in the table, the relative density of PAB decreased from 94.92% to 88.43% as the Bi_2_O_3_ content increased from 0 to 18%.

### 2.2. Drop-Hammer Test

**Figure 2:**
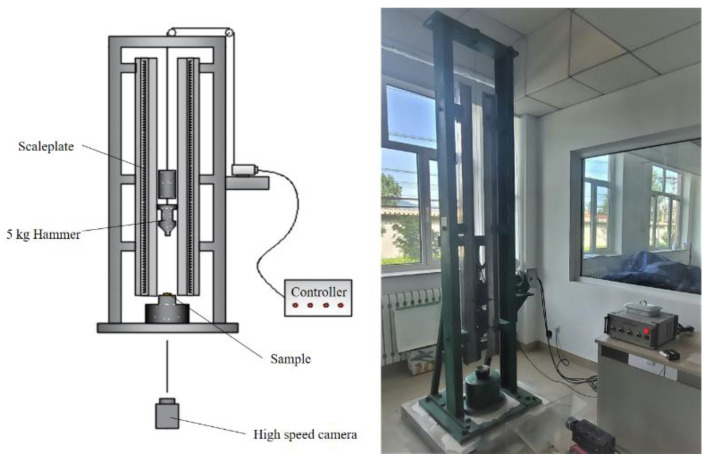
Schematic diagram of drop-weight test and site layout.

The drop-hammer test, also known as the drop-weight test, is a practical and straightforward impact test method. The response characteristics of PAB with different Bi_2_O_3_ contents under a low strain rate and long pulse impact can be compared with the help of a drop-hammer test setup. The mass of the drop hammer in the test was 5 kg, the measuring range was 0~150 cm, and the ambient temperature was 25 °C. The drop-hammer experimental setup is shown in Figure 2. 

The characteristic drop height *H*_50_ at which a 50% shock-induced chemical reaction occurs can characterize the impact sensitivity of the energetic material. The experiment was carried out using the up-and-down method [20]. Each material was tested 20 times, and the experiment interval was 5 cm. The characteristic drop height *H*_50_ can be calculated according to the equation:

(3) $$H_{50}=A+B\left(\frac{\sum \;{\mathrm{iC}}_{i}}{D}-\frac{1}{2}\right)$$

where *A* is the lowest falling height in the test, *B* is the experimental spacing, *i* is the level ordinal number of falling height, *C_i_* is the number of shock-induced chemical reactions occurring at a certain blasting height, and *D* is the total number of reactions occurring in the test.

To compare the energy release of PAB materials with different Bi_2_O_3_ contents, the same drop heights were used for all the tests. To more accurately record the firing behavior of the specimen, a high-speed camera was used to record the whole process at a frame rate of 10,000 frames/second.

### 2.3. Thermal Analysis

The physical or chemical reaction occurring in the sample during heating can be analyzed using the mass- and heat-change data. Therefore, a combined thermogravimetric–differential scanning calorimetric (TG-DSC) system was used to test the static thermal decomposition process of the ternary reactive materials. The physical and chemical changes in the material during the impact process can be reasonably inferred from the fixed thermal decomposition laws. The test apparatus was a differential scanning calorimeter (NETZSCH-STA449F3, Shanghai, China) and a thermogravimetric analyzer (NETZSCH-QMS403C, Shanghai, China).

Since the ratio of the mixture of the formulations has a negligible effect on the thermal reaction properties of the materials [21], we performed only two sets of experiments with 1#PA and 4#PAB. The two composites were heated linearly at 10 °C/min from 25 °C to 930 °C. The experiments were carried out under a high-purity argon atmosphere to avoid the effect of oxygen in the air, with an argon purge rate of 60 mL/min.

## 3. Results and Analysis

### 3.1. Energy Release Characteristics of PAB under Drop-Hammer Impact

#### 3.1.1. Shock-Induced Chemical Reaction Phenomenon

**Figure 3:**
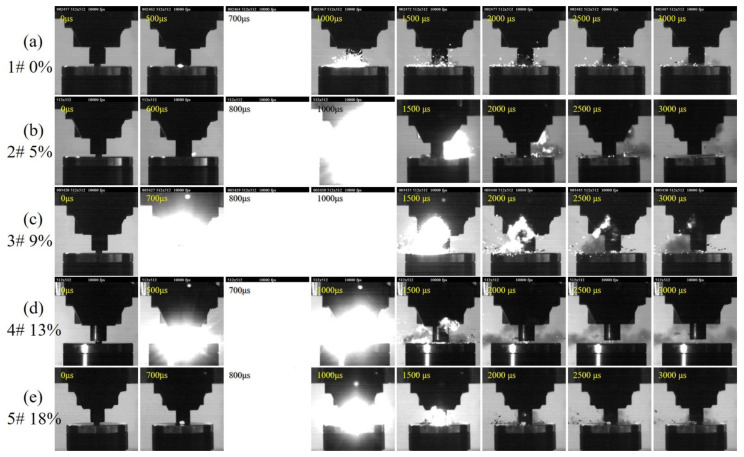
Shock-induced chemical reaction process images (height = 150 cm): (**a**) PA 1#; (**b**) 2#PAB-5%; (**c**) 3#PAB-9%; (**d**) 4# PAB-13%; (**e**) 5#PAB-18%.

When the drop height is 150 cm, PAB can react violently under the drop-hammer loading. Figure 3 shows the reaction process of PAB with different Bi_2_O_3_ contents under drop-hammer loading at 150 cm.

In the Figure 3, the first column of photographs is at 0 μs, wherein the falling hammer just touches the specimen. The second column of photographs depict the occurrence of ignition, and the third column are the photos with the largest and brightest flame range. These photos were taken at slightly different times depending on the Bi_2_O_3_ content. For specimens with different Bi_2_O_3_ contents, they all first undergo violent compression and deformation. The material rapidly fails, breaks up, and is accompanied by a large amount of debris scattering. After a short time, the reactive material is stimulated. The ignition occurs, and the flame spreads rapidly and transforms into a bright fire; the whole photograph shows a bright white color, then the flame gradually decreases, lasts for some time, then gradually disappears. An explosion sound accompanies the reaction process. After the reaction was extinguished, many PAB fragments were seen scattered in the air, and only a tiny part of the material reacted. The difference in the shock-induced chemical reaction phenomenon with different Bi_2_O_3_ contents is mainly reflected in the reaction duration.

**Figure 4:**
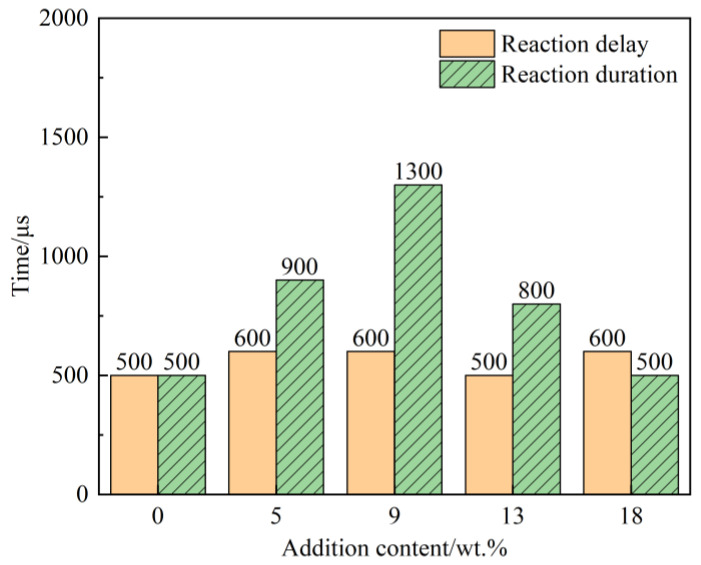
Shock-induced chemical reaction duration of PAB with different addition contents (height = 150 cm).

Figure 4 shows the reaction duration. The frame-rate of high-speed camera images is 10,000 frames, and the interval of each photograph is 100 μs. Considering the error, we think that the ignition of PABs with different Bi_2_O_3_ contents all occurred around 600 μs after loading. The Bi_2_O_3_ content had a greater influence on the shock-induced chemical reaction duration. When Bi_2_O_3_ was not added, the reaction was extinguished rapidly after ignition, and the reaction lasted 500 μs. As the Bi_2_O_3_ content increased from 0 to 9%, the reaction duration increased to 1300 μs, and the time of the violent reaction increased. It should be emphasized that only a tiny fraction of the material reacted during loading. Its reactivity and energy release rate remain low, but the experimental phenomenon still illustrates that the presence of Bi_2_O_3_ particles facilitates the reaction of the reactive material. 

For dense PTFE/Al reactive materials, the reaction is non-self-sustaining and difficult to propagate continuously [22,23,24]. When Bi_2_O_3_ particles are added, the Al/Bi_2_O_3_ thermite reaction will also be initiated after the PTFE/Al reaction occurs. The high burning rate of the Al/Bi_2_O_3_ reaction will release a large amount of heat in a short time to supplement the non-self-sustaining PTFE/Al reaction, thus prolonging the duration of the reaction. However, in PAB, the PTFE/Al reaction is still the primary reaction, and when the content of Bi_2_O_3_ particles is too much, the content of PTFE decreases. The heat released from its reaction decreases, which is not favorable for exciting the Al/ Bi_2_O_3_ reaction; therefore, when the content of Bi_2_O_3_ particles increases to 13%, the reaction duration becomes shorter.

#### 3.1.2. Impact Sensitivity

**Figure 5:**
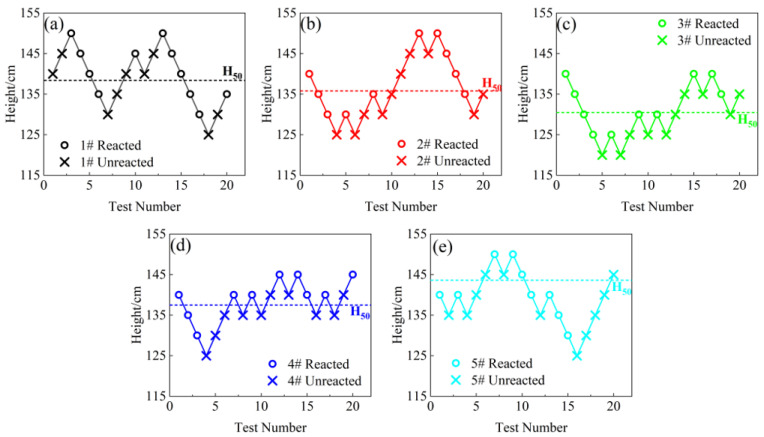
Drop-weight test results of PAB with different Bi_2_O_3_ contents: (**a**) PA 1#; (**b**) 2#PAB-5%; (**c**) 3#PAB-9%; (**d**) 4# PAB-13%; (**e**) 5#PAB-18%.

The results of the drop-hammer test for each group of materials can be seen in Figure 5, and the calculated characteristic drop heights *H*_50_ are listed in Table 3. 

The experimental results show that the content of Bi_2_O_3_ particles has a significant effect on the characteristic drop heights of PAB. As the particle content increases, the characteristic drop height of PAB first decreases, and then increases. When the content of Bi_2_O_3_ particles is 9%, the characteristic drop height is 130.5 cm, which is the lowest among the five formulations. When the content of Bi_2_O_3_ particles exceeds 13%, the characteristic drop height *H*_50_ starts to increase, and when the content of particles reaches 18%, the characteristic drop height *H*_50_ is higher than that of 1#PA.

PAB is a typical polymer-based granular composite with good plasticity when sufficient PTFE matrix can undergo large deformation under impact loading. As the particle content increases, the number of particles in the ternary composite increases. The PTFE matrix is non-polar, the surface energy is very low, and the interface between the particles and the matrix is only mechanically bonded, so its interfacial properties are very poor, resulting in the interfaces being a defect in the composite [25]. When the particle content increases, the area of interface increases considerably and its defects increase accordingly; moreover, deformation incongruity between the matrix and the filler particles becomes more severe during impact loading. The internal force is more likely to be concentrated in a small area, as it is more difficult for the impact force to be dispersed to the whole material system. At the same time, the friction between particles is intensified [18], which causes the composite to become brittle and more prone to cracking; moreover, more hot spots appear in the composite during the loading process, where reactions are more easily triggered. Therefore, the characteristic drop-height values of the ternary reactive materials all decrease when the content of Bi_2_O_3_ particles increases. As the particle content increases significantly, the content of the matrix decreases relatively, and the oxidant in the composite decreases. It is somewhat more difficult for both the PTFE/Al reaction and the Al/Bi_2_O_3_ reaction to occur, so the material sensibility starts to gradually decrease when the Bi_2_O_3_ content is higher than 10%.

#### 3.1.3. Ignition Delay

**Figure 6:**
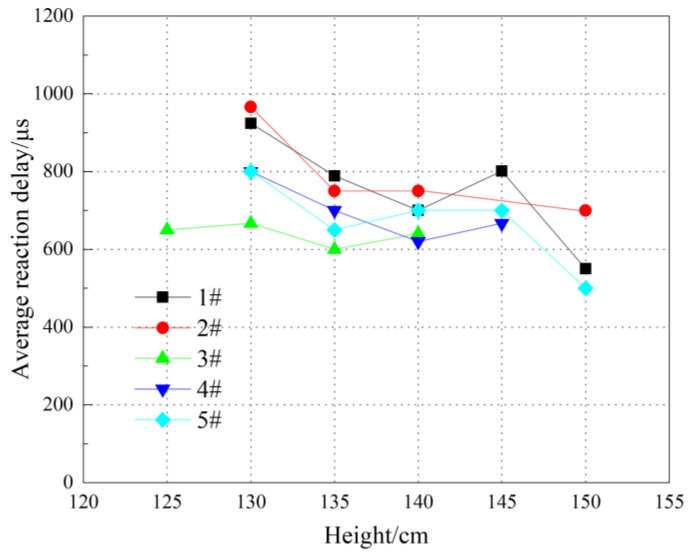
Reaction delay of reactive material under drop-hammer test.

The reaction delay time was defined as the time interval from 0 μs to the appearance of the ignition phenomenon, using 0 μs as the initial impact moment. The average reaction delay of several experiments at each drop height was calculated and shown in Figure 6. 

It is clear from Figure 6 that the reaction delay of each group is relatively less affected by the content of Bi_2_O_3_ particles. The reaction delay is distributed between 500 and 1000 μs, with most of them between 600 and 800 μs. The ignition delay shows a decreasing trend with the increase in drop height. This also implies that the ignition delay decreases with increasing loading strain rate, which is consistent with the results of previous studies [24]. Drop-hammer loading is a type of long-pulse loading, where the total loading time can reach several milliseconds. The stress value gradually increases in continuous loading, and the stress rises faster with time as the drop height increases. The specimens are rapidly and violently deformed in the early loading stage, and the stress slowly increases. After the specimen is impacted for 500–1000 μs, the stress begins to rise rapidly, and can even reach 1.5 GPa [26], which long-exceeds the stress ignition threshold of PAB [27]. Thus, ignition occurs.

### 3.2. Shape of the Specimen before and after the Reaction

#### 3.2.1. Macroscopic Appearance of Specimens

**Figure 7:**
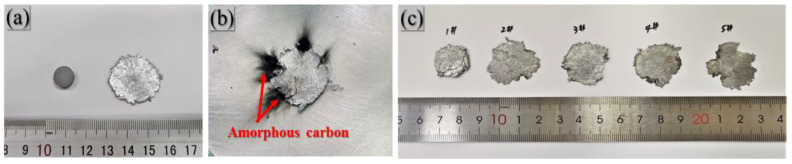
Specimen before and after drop-weight test: (**a**) unreacted specimen before and after drop weight test; (**b**) reacted specimen; (**c**) reacted specimen of different Bi_2_O_3_ contents.

PAB can react violently under the drop-hammer loading conditions, and an apparent fire and explosion sound accompany the reaction. The material overreacts, from reacting to unreacted, as the drop-hammer height decreases. Figure 7 shows the specimen’s morphology before and after the drop-hammer loading.

The specimens underwent great deformation under drop-hammer loading, and their strains could reach 0.8–0.9. The specimens without ignition remained relatively intact after loading, and the edges of the specimens were slightly rough. At the same time, multiple open-type cracks appeared at the edges of the specimens where ignition occurred, and apparent jet-like carbon black appeared at the corresponding positions of the cracks. The same phenomenon occurs in all ignition specimens in which black remnants could only be found in the mode I cracks. This phenomenon is also consistent with other research results [28]. The 1#PA specimen was thicker after loading, and the material broke in multiple places. The ductility of the specimen containing Bi_2_O_3_ particles was greatly improved, and the specimen strain increased.

#### 3.2.2. Microscopic Morphology of Specimens

**Figure 8:**
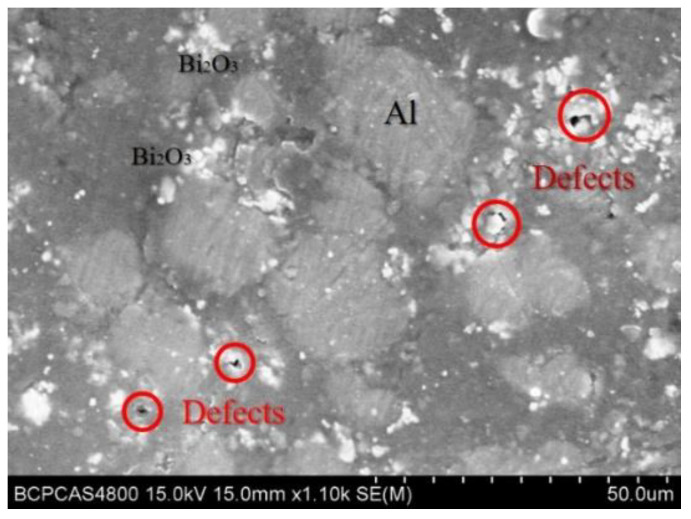
Typical SEM photographs of PAB.

Figure 8 shows the SEM images of the prepared 4#PAB specimens. After sintering, the PTFE matrix encapsulates the intact particles. In PAB, the PTFE matrix can cover the Al particles well, but some of the Bi_2_O_3_ particles are agglomerated and not well dispersed in the matrix, and there are holes between the Bi_2_O_3_ particles.

**Figure 9:**
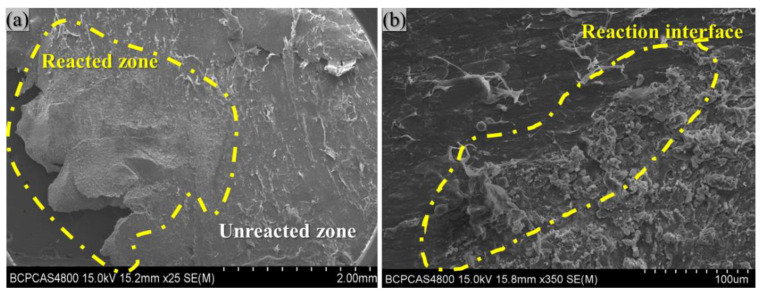
(**a**) Reacted specimen after drop-weight test; (**b**) reaction interface.

Figure 9 shows the SEM results at the reaction position of the collected specimens. As seen in Figure 9, the reaction interface is obvious, and the difference between the reacted and unreacted zone is visible. The Al particles and Bi_2_O_3_ particles are distributed more uniformly in the unreacted zone, and the specimen’s surface is flat and dense. The texture is very rough in the reaction zone, and the overall appearance is loose and porous. Many spherical protrusions, melted PTFE, and pores are visible in the matrix. 

**Figure 10:**
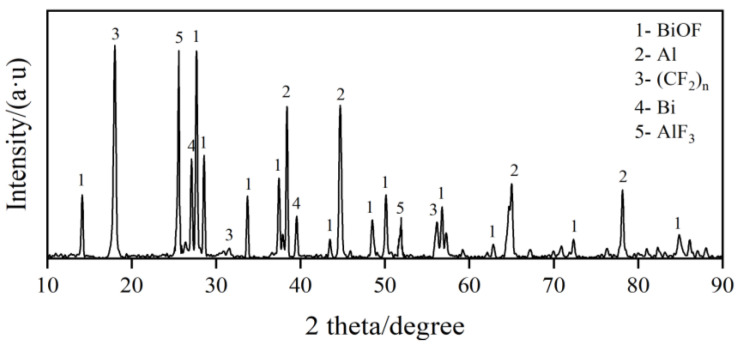
Micro-XRD of reacted zone.

The Micro-XRD results of the reacted zone of PAB are shown in Figure 10. For PABs, Al, PTFE, AlF_3_, Bi and BiOF are present in the reaction residue. The occurrence of reaction products such as Al_3_C_4_, AlF_3_, Bi and BiOF confirm the occurrence of a shock-induced chemical reaction.

### 3.3. Ignition and Reaction Mechanism of PAB under Drop-Hammer Impact

#### 3.3.1. Hot-Spot Formation

**Figure 11:**
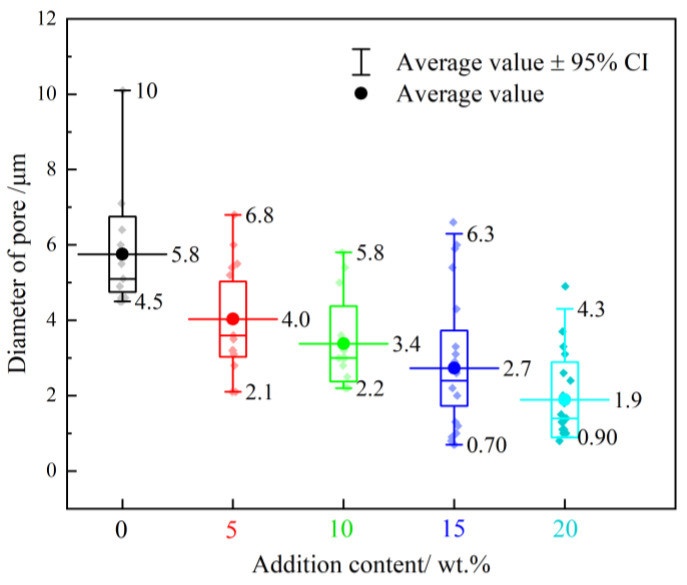
Box plot of hole diameter statistics.

Potential hotspot mechanisms for reacting reactive materials are pore collapse, crack generation and convergence, friction, plastic work, and shear band generation [29]. The sizes of the pores captured in the SEM images were counted, and the results are shown in Figure 11.

It is clear that the pore sizes have a specific distribution, and their average sizes are correlated with the ratios. The pores in the 1# PA without the addition of Bi_2_O_3_ are relatively large, and their average diameter size is 5.8 μm. In formulations 2–5#, the average size of the pores in the material decreases with the increase in the Bi_2_O_3_ particle content. It should be noted that although the average size of pores decreases, the number of pores increases substantially and the composite becomes relatively more loose and porous, as shown in Table 2. The pores in each group are tiny, and their average size is only a few microns. Additionally, the test is performed using drop-hammer loading at a low impact velocity and loading strain rate. Thus, we believe that the heat generated by the viscoplastic work during the collapse of the pores has a negligible effect on the ignition of the reactive material.

**Figure 12:**
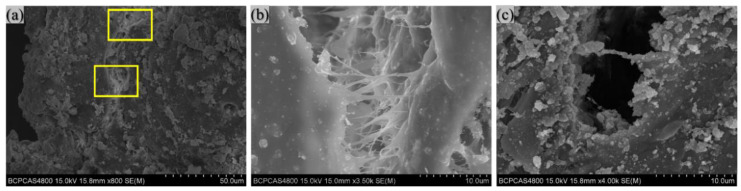
SEM photograph of crack: (**a**) crack; (**b**) edge of the crack; (**c**) tip of the crack.

Subsequently, microscopic observations were made of the cracks in the collected specimens, as in Figure 12. There are cracks with a width of about 10 μm in the reaction region of PAB. At the opening of the crack in Figure 12b, there are evident traces of melted PTFE, Bi_2_O_3_ particles are present at the edge of the crack, and numerous PTFE microfibers with a diameter of less than 1 μm are visible in the crack; furthermore, some microfibers have been pulled off. At the tip of the crack in Figure 12c, a few microfibers and more spherical protrusions are apparent evidence that reactions have occurred.

When impacted by the falling hammer, the material is first violently deformed, the particles are extruded and rubbed against each other during the deformation, the pores in the material collapse and close, and the particles and the PTFE matrix are de-bonded. A large amount of damage and micro-cracks appear inside the material. As the loading continues, the stress increases rapidly, the specimen becomes more extensive and thinner, the PTFE microfibers can no longer compensate for the development of micro defects, and the micro-cracks expand further. Many internal micro-cracks grow at an accelerated rate and proliferate into cracks. The rapidly expanding crack tip produces immense fracture energy, resulting in a temperature rise in the local area. The temperature rise—determined by the plastic work, the size of the local plastic deformation region, and the crack velocity which can reach 300–1000 °C [30]—is sufficient to cause the reaction. This explains why there is apparent evidence of a reaction at the tip of the crack in Figure 12c.

The experimental results show an optimal range (around 9%) of Bi_2_O_3_ content in PAB. When the Bi_2_O_3_ content is in this optimal range, the deformation incompatibility between the PTFE matrix and Al particles and Bi_2_O_3_ particles during impact loading, and the particle-to-particle friction, will intensify the local stress concentration in the reactive material; this leads to the reactive material being more prone to cracking and the formation of more hot spots.

#### 3.3.2. Thermal Decomposition Process

**Figure 13:**
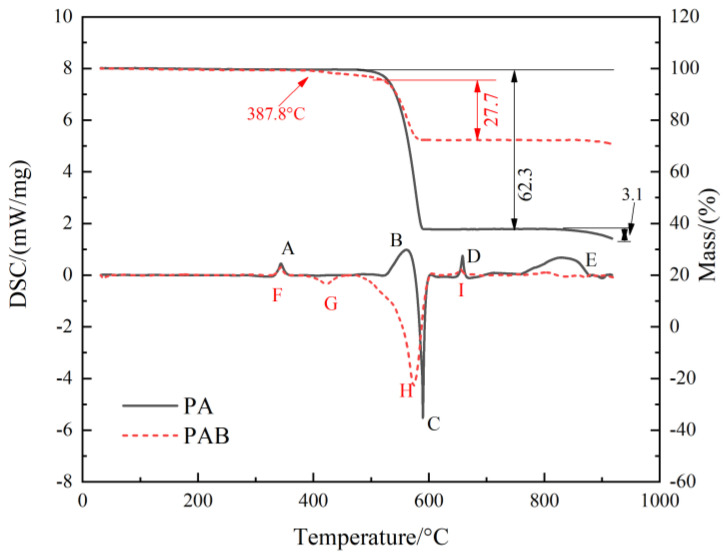
TG-DSC curve of PA and PAB.

The physicochemical change patterns of the reactive materials obtained by the DSC-TG test under static linear heating can effectively guide understanding of the complex reaction process of PAB during local heating under impact. We perform tests under an argon atmosphere to investigate the rapid and short shock-induced chemical reactions that occur inside the composite. Figure 13 shows the TG-DSC curves of PTFE/Al and PAB; the peak shape upward represents the heat absorption, and downward represents the heat release. The relevant parameters and physical and chemical changes in each peak are listed in Table 4. 

There are five peaks in the DSC curve of PA, among which peak C is exothermic, and the other four peaks are all heat-absorption peaks. The appearance of peak A is attributed to the melting heatabsorption of the PTFE matrix. The heat-absorption peak (B) appears at 522 °C along with the sample mass loss, which indicates that the heat-absorption peak is due to the decomposition of PTFE by thermal depolymerization, and CF_2_ may be the main product in this phase [31]. The exothermic peak (C) occurs at 564 °C and ends at 605 °C, during which PTFE-Al shows significant weight loss, reaching 62.3%. This is due to the strong oxidative decomposition products of PTFE reacting exothermically with the aluminum powder in the material to produce AlF_3_, carbon and Al_4_C_3_. Peak D is the melting endothermic peak of the residual Al powder. After 750 °C, peak E appears to have a mass loss of about 3.1%, which can be attributed to the sublimation of AlF_3_ at about 800 °C [32].

The DSC and TG curves of PAB have some differences from those of the PAs. Only four peaks appear in the DSC curve of PAB. Peaks F and I are similar to peaks A and D in the DSC curves of PAs, which are the melting peak of the PTFE matrix and Al, respectively. Peak G appears with weight loss, and the first peak on the DSC curve of Bi_2_O_3_/Al super thermite appears at 480 °C [15]; thus, peak G comes from the reaction of PTFE with solid Bi_2_O_3_ at around 385 °C. As the temperature continues rising to 480–550 °C, the alumina on the Al surface breaks up in the phase transition, and some of the active Al is exposed and comes into contact with Bi_2_O_3_ particles. Hence a solid–solid phase aluminothermic reaction occurs, generating gas Bi and slight weight loss. When the temperature gradually increases to about 520 °C, the weight loss curve suddenly becomes steeper, indicating that PTFE pyrolysis occurs and it starts to participate in the violent exothermic reaction. Therefore, the appearance of peak H should be the superposition of the Al/Bi_2_O_3_ reaction, PTFE pyrolysis, and the PTFE/Al reaction. The DSC curve of PAB does not show a similar peak to the E peak in the DSC curve of PA, which also confirms that the peak H is a multiple-reaction peak: the Al/Bi_2_O_3_ in front may consume Al, less Al reacts with PTFE, and less AlF_3_ is generated, which is not enough for sublimation to form the heat-absorption peak during the heat treatment.

PAB has improved energy release compared with PA, which specifically enhances the material’s reactivity. At the same time, the start temperature of the reaction decreases, which may lower the excitation threshold of the reactive material and improve the degree of reaction, favoring the application of the reactive material.

#### 3.3.3. Microscopic Morphology of Reaction Products

**Figure 14:**
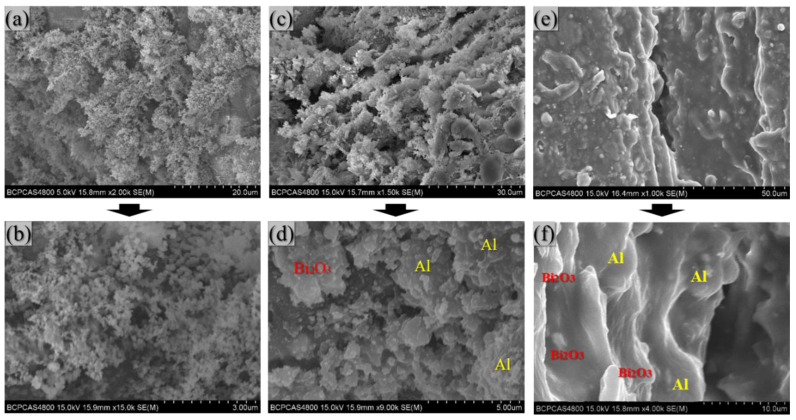
SEM photos of remnant: (**a**,**b**) fully reacted zone; (**c**,**d**) partial reacted zone; (**e**,**f**) melt zone.

The reacted zone in PAB shows several different morphologies, and Figure 14 shows the microscopic morphology of some specific regions. The surface morphology of PAB changes significantly after the reaction and is no longer a dense structure.

In Figure 14a, many smoke-like structures exist on the loose and porous surfaces. Figure 14b shows a further magnified observation. The smoke-like structure comprises hundreds of spherical particles with tens of nanometers in diameter, forming chain, cluster, and dendritic systems. This is because the AlF_3_ and Bi produced by the reactions of PTFE/Al and Al/Bi_2_O_3_ are both gaseous, and when the gaseous and condensed products diffuse to the particle surface, they will condense into a loose, porous structural material. At the same time, the alumina fumes produced by the reaction of Al/Bi_2_O_3_ and the PTFE/Al reaction product, carbon, will also condense toward the particle surface, and the microscopic morphology of the combustion products are all quasi-spherical particles with a diameter of several tens of nanometers [33,34,35]. The gaseous and solid products form the reaction product morphology in, as seen in Figure 14a,b.

In Figure 14c, the morphology is mainly spongy and contains many pores. There are still apparent spherical Al particles and Bi_2_O_3_ particles in the image of Figure 14d when observed at magnification. In this region, only part of the reaction takes place. There are pores due to the reaction, relatively dense multiphase reaction products, and also intact active particles. The pores provide room for the growth of spherical crystals during the cooling of the PTFE, thus creating this complex morphology.

In Figure 14e, the surface is very smooth with soft contours. There are very few holes in the matrix and between the matrix and the particles, and PTFE is dominated by band crystallization, which is denser in the microstructure. Figure 14 shows a partial enlarged view. Figure 14f and the energy spectrum results show that the Al particles and Bi_2_O_3_ particles are covered by the matrix. In this region, only the physical change in the PTFE matrix occurs, and few chemical reactions take place.

#### 3.3.4. Ignition and Reaction Propagation Process

**Figure 15:**
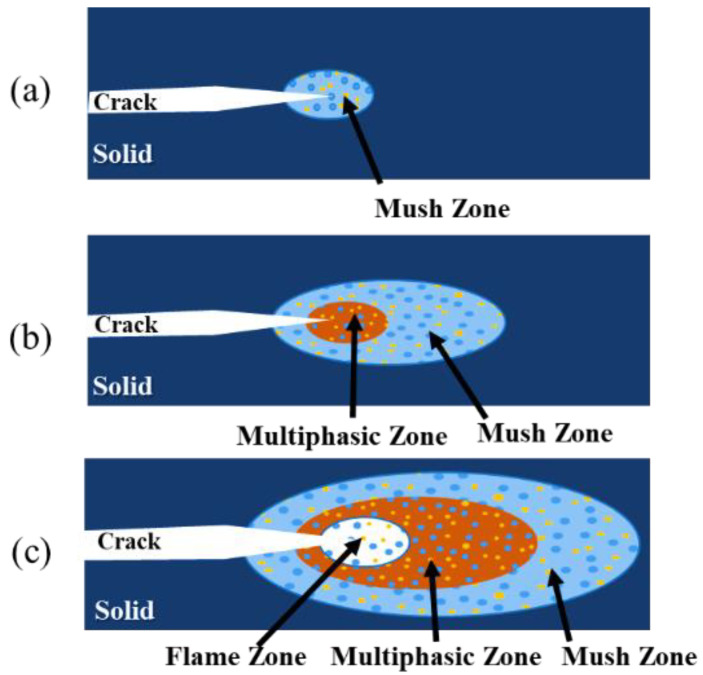
Ignition process of PAB: (**a**) Local PTFE melts to form mush zone; (**b**) Multiphasic zone is formed; (**c**) Flame zone appears.

Combining the physicochemical changes with the microscopic product morphology, we can speculate that the ignition process of PAB is approximately as follows (Figure 15 shows the schematic diagram).

When the crack tip exotherm generates a very high heating rate locally, a violent temperature rise occurs in the local area, and the solid phase material starts to absorb energy. The local PTFE starts to melt at a certain point, forming a mush zone containing Al and Bi_2_O_3_ solid particles, as shown in Figure 15a. Figure 14e,f shows the microscopic morphology. 

When the liquid phase forms, the solid–liquid boundary begins to expand continuously by heat conduction and thermal radiation, and the temperature in the mush zone keeps rising. When the temperature rises to 385–422 °C, the reaction between liquid-PTFE and solid-Bi_2_O_3_ occurs, and when the temperature rises to 472 °C, the solid-Al/solid-Bi_2_O_3_ reaction begins. When the temperature reaches 520 °C, the thermal decomposition of the substrate begins to accelerate. All three physicochemical changes will produce gas and bubbles in the region, thus forming a solid–liquid–gas three-phase zone, such as the multiphasic zone in Figure 15b. Figure 14c,d shows the reaction products’ microscopic morphology.

As the temperature increases, a large amount of the PTFE matrix decomposes into strongly oxidizing C_2_F_4_ molecules, exposing the Bi_2_O_3_ particles and a large amount of Al particles to the strongly oxidizing gas atmosphere. Bi_2_O_3_ particles and Al particles undergo surface chemical reactions and release heat while exchanging heat with the oxidizing gas, and the temperature of the particles increases continuously. Then, the Al particles ignite and burn violently in the strong oxidizing gas, as shown in the flame zone in Figure 15c, and the microscopic morphology of the reaction zone in Figure 14a,b.

**Figure 16:**
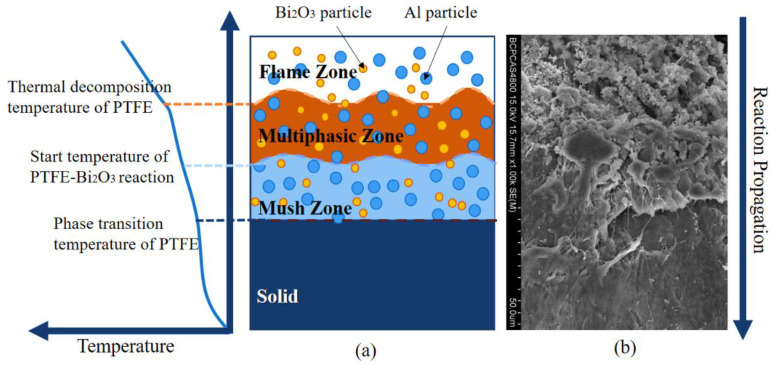
(**a**) Reaction propagation of PAB; (**b**) SEM of PAB reaction propagation.

This ignition process explains the reaction propagation process of PAB. Figure 16a shows the reaction propagation process of PAB. 

In the flame zone, the Al particles burn violently in the strongly oxidizing region and release a large amount of energy. The temperature is gradually propagated by heat conduction and thermal radiation, and the greater the distance from the reaction region, the lower the temperature is. In the multiphasic zone, which is close to the flame zone, PTFE decomposes due to heat absorption. However, the exothermic reaction of molten PTFE/Bi_2_O_3_ and Al/Bi_2_O_3_ replenishes the consumed energy so that the temperature can continue to propagate by radiation and form a mush zone of molten PTFE wrapped with Al particles and Bi_2_O_3_ at a further distance. Due to the heat absorption from the PTFE matrix melting, the expansion of the mush zone can only rely on the heat transferred from the reaction area or the local hot-spot temperature rise. The melting of the high-content PTFE matrix requires a large amount of heat absorption, so developing the interface between the mush zone and the solid phase is relatively difficult. Therefore, it is challenging for the reaction in PAB to propagate in a self-sustainable way, and only a tiny fraction of the reactive material reacts. 

Figure 16b shows the microscopic image at the reaction interface of the specimen, which can show the reaction propagation process from top to bottom as a fully reacted region, partially reacted region, molten region, and unreacted region.

With a proper amount of Bi_2_O_3_, the reaction between molten PTFE and Bi_2_O_3_, and the aluminothermic reaction between Al/Bi_2_O_3_, will occur more easily in the multiphasic zone. The large amount of energy released from these two exothermic reactions will facilitate the diffusion in the multiphase zone, thus promoting the propagation of the overall reaction in PAB. Therefore, in the drop-hammer test, the reactivity of PAB gradually increases when the Bi_2_O_3_ content increases from 0% to 9%. However, when the Bi_2_O_3_ content is too much, the reaction of molten PTFE and Bi_2_O_3_, and the aluminothermic reaction between Al/Bi_2_O_3_, consume the Al and oxidizing C_2_F_4_ molecules in advance; this makes the content of the components involved in the C_2_F_4_/Al reaction, as well as the reaction and energy-release ability of PAB, decrease.

#### 3.3.5. Ignition of Aluminum Particles

For aluminum particles, the ignition mechanism is dominated by the heating rate. During the mechanical impact of the drop hammer in this experiment, the mechanical load generated high stress and a high strain rate, which converted the mechanical energy into heat energy with a heating rate of up to 10^4^ K/s [36]. In general, for micron scale particles and nanoparticles at slow heating rates, oxidation is expected to occur by diffusion ignition mechanism [37]. This mechanism suggests that the huge pressure difference inside and outside the aluminum oxide shell accelerates the mass transfer of oxide and metal atoms between the metal core and the oxide shell. The ignition process of Al particles is divided into two stages. Before Al melting, oxide diffuses through a growing oxide shell and reacts with aluminum in a slow oxidation reaction; when the temperature is higher than the aluminum melting point, the oxidation reaction is accelerated, atoms diffuse and react at the same time, and the diffusion speed is constantly accelerated by the huge pressure difference inside and outside the oxide layer generated by the melting of aluminum. The melting of the oxide layer accelerates the oxidation rate [38,39].

**Figure 17:**
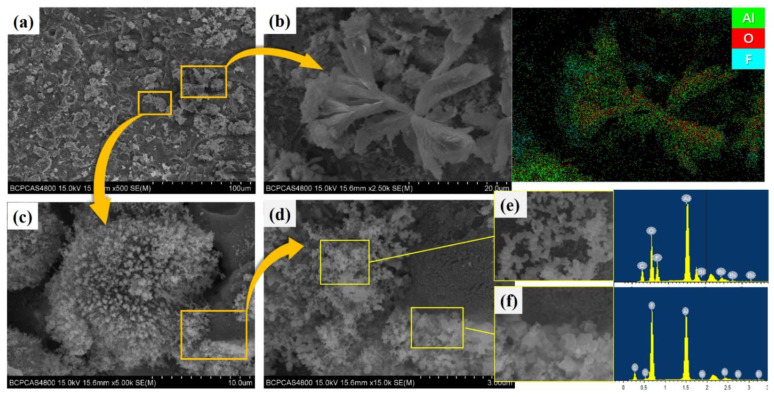
SEM photograph of reaction remnant: (**a**) Al protrusion embedded in the matrix at the reaction interface; (**b**) ruptured Al_2_O_3_ shell; (**c**) reaction products on the surface of Al particles; (**d**) reaction products; (**e**) Al_2_O_3_ smoke; (**f**) AlF_3_ crystal products.

Figure 17 shows SEM photos of the typical morphology of aluminum particles after reactions. A large number of spherical particles are present on the reaction surface, half of which are exposed, and the exposed surface is very rough, with some protrusions. In contrast, the surface of the matrix is much smoother. The energy spectrum results show that the spherical particles are aluminum. There are two main structures of aluminum particle surface protrusions: smoke formed by the joining of multiple spherical particles of several tens of nanometers in size, or crystals (see Figure 17e,f). There is a large number of reaction products on the surface of aluminum particles and almost no reaction products on the surface of the matrix, which confirms that the ignition of Al particles is consistent with the diffusion ignition mechanism. Moreover, the start temperature of PAB is much lower than the melting temperatures of Al (660 °C) and Bi_2_O_3_ (820 °C), which also indicates that the ignition of Al particles is more consistent with the diffusion ignition mechanism.

In addition, crack expansion in the reactive material after impact may also tear the inert alumina shell of the Al particles, exposing the internal active aluminum. This is evidenced by several photographed broken alumina shell layers, as shown in Figure 17b.

In PAB, Al participates in two reactions. A solid–solid reaction between Al-Bi_2_O_3_ particles at 472–520 °C, and a fluorination reaction with strongly oxidizing C_2_F_4_ gas between 520–574 °C. In the reaction of Al/Bi_2_O_3_ particles, the oxide layer of Al will undergo a crystalline change from amorphous Al_2_O_3_ to γ-Al_2_O_3_ in the crystalline phase when the temperature rises above 480 °C [40]. The density difference between the two phases of Al_2_O_3_ will make the oxide layer shrink and thin during the phase change. The ions in bismuth oxide can diffuse rapidly into the Al_2_O_3_ shell layer and trigger the solid–solid reaction of Bi_2_O_3_ and Al. This aluminothermic reaction will provide a large amount of energy for the local accelerated temperature rise. In the Al/PTFE fluorination reaction, BiF_3_, the product of the Bi_2_O_3_/PTFE reaction, can catalyze the thermal decomposition of PTFE to some extent, so PTFE starts to accelerate the pyrolysis in advance. The strongly oxidizing C_2_F_4_ gas will react with the hydroxyl radicals on the surface layer of Al particles and contact the bare active aluminum. As a result, the Al particles ignite violently in the strong oxidizing atmosphere and release a large amount of energy.

The melt-dispersion mechanism is not applicable here. The key to the melt-dispersion mechanism is that the aluminum oxide shell passivates the Al particles, and the shell is free of defects, which requires the shell to possess a certain thickness and strength. While in the reaction of PAB, vital oxidizing C_2_F_4_ molecules can react with the hydroxyl radical of Al_2_O_3_ and destroy the oxide layer. It is also difficult to form high pressure in the micron Al particles to make the active aluminum break up and fly apart. The active aluminum core will gradually be oxidized by the traditional diffusion mechanism.

From the ignition of Al particles, the presence of Bi_2_O_3_ in PAB can effectively enhance the ability of Al to participate in the reaction, thus improving the energy-release characteristics of the reactive material.

## 4. Conclusions

In this paper, the energy release characteristics of PAB with different Bi_2_O_3_ contents under the low-speed impact of a drop-hammer test were investigated, and the influence law of Bi_2_O_3_ content in PAB on the impact reaction characteristics was studied. The ignition and reaction mechanism of PAB under the drop-hammer impact was analyzed with the help of scanning electron microscopy to observe the microscopic morphology before and after the test and DSC thermal analysis. The main conclusions are as follows.

(1) At a drop height of 150 cm, PABs can react vigorously under drop-hammer loading. The ratio influenced the reaction duration and intensity. As the Bi_2_O_3_ content increased to 9%, the reaction duration increased and the reactivity increased, and when the content continued to increase, the reaction duration decreased and the intensity of the reaction decreased. When the Bi_2_O_3_ content was 9%, the reaction was the most intense, and the duration was the longest at 1300 μs. With the increase in Bi_2_O_3_ content in the system, the sensitivity of the material first increased, and then fell. When the Bi_2_O_3_ content was 9%, the highest impact sensitivity was achieved with a characteristic drop height *H*_50_ of 130.5 cm.
(2) Under impact loading of the drop hammer, ignition occurs at the tips of open cracks. PAB undergoes several physicochemical processes such as PTFE melting, a PTFE/Bi_2_O_3_ reaction, an Al/ Bi_2_O_3_ reaction, molten PTFE pyrolysis, and a C_2_F_4_/Al reaction during the local temperature rise. The presence of Bi_2_O_3_ results in a lower reaction onset temperature of the reactive material, a lower excitation threshold of the reactive material, and a higher degree of reaction, improving the overall energy release of the reactive material.
(3) The ignition process and propagation process in PAB can be roughly divided into three stages: local PTFE melting (mush zone); the reaction of the active component and thermal decomposition of the matrix to produce gas (multiphase zone); and violent combustion of Bi_2_O_3_ and Al particles in strong oxidizing C_2_F_4_ gas (flame zone). With a appropriate amount of Bi_2_O_3_, the reaction of molten PTFE and Bi_2_O_3_ and the thermit reaction between Al/Bi_2_O_3_ will occur more easily in the multiphasic zone; this will release a large amount of energy and promote diffusion in the multiphasic zone, thus promoting the propagation of the overall reaction and improving the reactivity of PAB.

## Figures and Tables

**Table 1:** Details of the formulation.

Formulation Codes	Al/Bi_2_O_3_ Additive Content/(wt.%)	Content/(wt.%)			Theoretical Density /(g∙cm^−3^)	Theoretical Calorific Value/(kJ∙g^−1^)
		PTFE	Al	Bi_2_O_3_		
1	0	74	26	-	2.32	8.42
2	5	70	25	5	2.41	8.11
3	10	67	24	9	2.48	7.79
4	15	63	24	13	2.57	7.48
5	20	59	23	18	2.68	7.16

**Table 2:** Density data of PABs with different Bi_2_O_3_ contents.

Formulation Codes	Bi_2_O_3_ Content/(%)	Theoretical Density /(g∙cm^−3^)	Actual Density /(g∙cm^−3^)	Relative Density /(%)	Porosity Ratio /(%)
1	0	2.32	2.19	94.92	5.08
2	5	2.41	2.27	93.59	6.41
3	9	2.48	2.32	92.77	7.23
4	13	2.57	2.33	90.66	9.34
5	18	2.68	2.38	88.43	11.67

**Table 3:** *H*_50_ of PAB with different Bi_2_O_3_ contents.

Bi_2_O_3_ Content/(%)	Characteristic Drop Height *H*_50_/(cm)
0	138.4
5	135.8
9	130.5
13	137.5
18	143.6

**Table 4:** Heat absorption and release of peaks in DSC curve.

Formulation	Peak	Start Temperature /(°C)	Peak Temperature /(°C)	End Temperature /(°C)	Energy Release /(J/g)	Physicochemical Changes
PTFE-Al	A	325	343	360	−36.0	Melting of PTFE
	B	522	561	576	−243.8	Decomposition of PTFE
	C	564	590	605	344.4	PTFE/Al reaction
	D	645	658	668	−35.4	Melting of Al
	E	755	825	877	−380.4	Sublimation of AlF_3_
PTFE-Al-Bi_2_O_3_	F	329	342	354	−23.6	Melting of PTFE
	G	385	422	446	61.4	PTFE/Bi_2_O_3_ reaction
	H	472	574	602	1042.2	Al/Bi_2_O_3_ reaction, Decomposition of PTFE, and PTFE/Al reaction
	I	642	656	661	−7.5	Melting of Al
